# Using a participatory method to test a strategy supporting the implementation of a state policy on screening children for adverse childhood experiences (ACEs) in a Federally Qualified Health Center system: a stepped-wedge cluster randomized trial

**DOI:** 10.1186/s43058-021-00244-4

**Published:** 2021-12-20

**Authors:** Monica Perez Jolles, Wendy J. Mack, Christina Reaves, Lisa Saldana, Nicole A. Stadnick, Maria E. Fernandez, Gregory A. Aarons

**Affiliations:** 1 Suzanne Dworak-Peck School of Social Work, Affiliate Gehr Family Center for Health Systems Science, University of Southern California, Los Angeles, CA USA; 2 Department of Population and Public Health Sciences, Keck School of Medicine, University of University of Southern California, Los Angeles, CA USA; 3 The House Institute Foundation, Los Angeles, CA USA; 4 Oregon Social Learning Center, Eugene, OR USA; 5 Child and Adolescent Services Research Center, San Diego, CA USA; 6 Department of Psychiatry, University of California San Diego, La Jolla, CA USA; 7 Altman Clinical and Translational Research Institute Dissemination and Implementation Science Center, University of California San Diego, La Jolla, CA USA; 8 Center for Health Promotion and Prevention Research, University of Texas Health Science Center at Houston School of Public Health, Houston, TX USA

**Keywords:** Policy implementation, Adverse childhood experiences (ACEs), Implementation mapping, Exploration, Preparation, Implementation, Sustainment (EPIS) Framework, Federally qualified health centers, Community engagement

## Abstract

**Background:**

Adverse childhood experiences (ACEs) are potentially traumatic events occurring before age 18, such as maltreatment or exposure to violence. ACE screening is increasingly recommended to prevent and address physical and mental health conditions associated with ACEs. To promote ACE screening uptake, the state of California issued the “ACEs Aware” policy that provides Medicaid reimbursement for ACE screening annually for child primary care visits. However, policy directives alone often do not translate into effective screening efforts and greater access to care. Few rigorous studies have developed and tested implementation strategies for ACE pediatric screening policies. This study will fill this gap by testing a multifaceted implementation strategy in partnership with a Federally Qualified Health Center (FQHC) system serving low-income families in Southern California to support the ACE Aware policy.

**Methods:**

We will use Implementation Mapping, with study process and consideration of determinants and mechanisms guided by the EPIS framework, to co-create and refine an implementation strategy. The proposed strategy is comprised of online training videos, a customized algorithm and use of technology to improve workflow efficiency, implementation training to internal FQHC personnel, clinic support and coaching, and written implementation protocols. A hybrid type 2, stepped-wedge cluster randomized trial design with five primary care clinics will test whether a multifaceted implementation strategy improves (a) fidelity to the ACE screening protocol, (b) reach defined as the proportion of eligible children screened for ACEs, and (c) the impact of the ACE policy on child-level mental health referrals and symptom outcomes. The study will use mixed methods with data to include electronic health records, surveys, and interviews with clinic personnel and caregivers.

**Discussion:**

This study is designed to increase the capacity of FQHCs’ inner context to successfully implement an outer context-initiated ACE policy designed to benefit pediatric patients. It capitalizes on a rare opportunity to use a co-creation approach to develop, adapt, refine, and pilot test an implementation strategy to maximize the impact of a new state-wide policy intended to improve ACE assessment and subsequent care to improve child health, particularly those from underserved communities.

**Trial registration:**

Trial # NCT04916587 registered at ClinicalTrials.gov on June 4, 2021.

Contributions to the literature

- We contribute to policy implementation by testing a comprehensive and tailored strategy supporting the implementation of a 2020 state policy promoting pediatric screenings to address potential risk for toxic stress and trauma associated with adverse childhood experiences.
- We contribute methodologically by demonstrating how Implementation Mapping can be combined with a process and determinant framework (i.e., EPIS) to engage researchers, healthcare managers, implementers, and end-users in a collaborative process.
- We address a literature gap by focusing on increased implementation capacity within a neglected complex health system—FQHC clinics serving low-income racial and ethnic minority families in diverse geographic settings.

## Background

Adverse childhood experiences (ACEs) refer to potentially traumatic events occurring before age 18, such as maltreatment, family separation, and exposure to violence [1, 2]. ACEs are pervasive, with 45% of children experiencing at least one ACE and 10% experiencing three or more ACEs, placing them at high risk for negative life outcomes [3]. Over 60% of Californians experienced at least one ACE before age 18 [3, 4]. ACEs are more prevalent among minority and immigrant communities due to exposure to poverty, discrimination, community violence, natural disasters, and refugee experiences [3]. When unaddressed, ACEs are associated with higher prevalence of post-traumatic stress disorder, depression, and suicide [5] as well as chronic physical conditions such as asthma and cardiovascular disease [4]. Earlier detection can inform relevant referrals, follow-up, ACEs prevention, and mitigate costs for more acute or long-term care [6–8].

Early screening for ACEs is a priority and increasingly recommended within primary care across the USA because ongoing exposure to ACEs can lead to toxic stress, which in turn can negatively impact brain functioning, physical health, school performance, and physical and mental health in later years [9–11]. Screening tools for early detection of healthcare needs are part of national quality improvement guidelines in line with the American Academy of Pediatrics recommendations [12]. Yet, ACE screening seldom occurs in pediatric primary care during well-child visits [13–15]. To facilitate widespread uptake of ACE screening, the Surgeon General of the state of California issued an ACE screening policy. Starting January 2020, the state’s Medicaid health care program provides reimbursement to primary care settings ($29) [16] for implementing ACE screenings annually during well-child visits to the Medicaid population [17, 18]. This ACE policy is supported by an annual budget of $40.8 million [19].

Despite significant investment in California and nationwide [20, 21], increased screening efforts often do not translate into greater access to care for children [22–24] and may even exacerbate health care access disparities by increasing stigma and reinforcing a deficit view of marginalized groups [25, 26]. Emerging interest in ACE screenings nationwide has pointed to a critical need to develop and test implementation strategies suited for pediatric screenings [26–29]. To address this need, this study will tailor and test a multicomponent implementation strategy to support effective implementation of universal ACE screening by FQHC system and responsive to a statewide policy in California. FQHCs serve as a healthcare safety net serving low-income populations with complex healthcare needs. The proposed strategy is comprised of training videos for ACE screeners, implementation coaching to support clinics as they adopt screening protocols, use of a validated clinical tool (i.e., Pediatric Symptoms Checklist or PSC), and an ACE algorithm to integrate and score these multiple sources of information.

This study will refine and test this implementation strategy and examine its impact on the fidelity and reach of ACE screening (implementation outcome) and resulting care linkage and mental health symptoms (clinical outcomes). The study capitalizes on a strong partnership with a Federally Qualified Health Center (FQHC) system in Southern California that adopted the ACE policy in August 2020. FQHCs serve the most vulnerable and low-income individuals and families in the United States. The partner FQHC has multiple diverse clinic locations and serves patients at high risk of ACEs due to poverty, systemic racism, exposure to violence, and migration-related trauma.

FQHC settings present an ideal context for implementation of the ACEs Aware policy, with a patient population at high risk of ACEs given the complex health and social needs of children and their families, and location in under-served geographic areas [30]. In a recent study of a large FQHC, 84.8% of patients reported experiencing at least one ACEs and 49.1% at least four ACEs [30]. Yet, FQHCs face multiple challenges in implementing new practices, including patient screenings, due to lack of infrastructure to foster efficiency and cost-effectiveness while limiting burden on an already strained workflow and workforce [31, 32]. The partner FQHC for this study is one of the largest systems in the country with over 200,000 total patients served annually [33]. In 2019 alone, 6345 patients ages 0–17 years old received care across 16 clinics. This FQHC system serves three counties covering frontier, rural, mid-urban, and urban areas.

## The California ACEs Aware policy

The policy goal is to “equip providers with training and clinical protocols to screen children and adults for ACEs, detect ACEs early, and connect patients to interventions, resources, and other support to improve patient health and well-being” [18]. In 2020, the state of California implemented a fee-for-service health policy by providing a financial incentive to Medicaid-serving clinics of $29 reimbursement for every child screened for ACEs annually [19]. The state-reimbursable ACE screening requires clinics to (a) complete a 2-h on-line provider training in the administration and interpretation of ACE screening, (b) use the Pediatric ACEs and Related Life-events Screener (PEARLS) tool, (c) use an ACE-associated health conditions checklist (e.g., asthma, allergies, anxiety, depression), and (d) complete a wellness exam [18]. The primary care provider uses a workflow provided by the state [34] to score each source of information to determine a final child risk score and provide follow-up education to the family [18].

This paper describes a study protocol for a hybrid effectiveness-implementation (type 2), using a stepped-wedge cluster randomized trial (SW-CRT) [35]. We selected the SW-CRT design because it accounts for calendar time and contextual (e.g., systems level) changes, both of which are relevant when conducting research in dynamic clinical settings [36].

## Conceptual framework

**Fig. 1:**
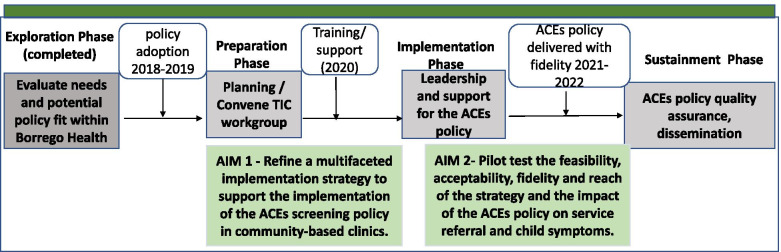
ACE study timeline across the phases of the epis framework

Implementation Mapping will be used to guide the development and tailoring of the implementation strategy and to inform its evaluation (assessment of implementation and clinical outcomes across the implementation and the sustainment phases). We will use all phases of the Exploration, Preparation, Implementation, Sustainment (EPIS) framework [37] to inform the planning and implementation process (Fig. 1).

The exploration phase occurred in 2018 with the FQHC identifying patient needs. The grant proposal was co-developed with the FQHC during this phase. The preparation phase started in 2019, convening a Trauma Informed Care (TIC) workgroup at the FQHC system. In early 2020, the partner FQHC promoted the ACE policy by requiring service providers (i.e., Physicians MD, Doctor of Osteopathic Medicine, Physician Assistants, and Nurse Practitioners) to complete the state-sponsored ACE 2-h training. The FQHC is currently preparing their electronic medical record (EMR) system to guide provider entry of ACE screening data, automate scoring, and track screening-related outcomes. This study focuses on the development and evaluation of a more rigorous strategy for implementation of the ACE policy.

The goal of aim 1 developmental phase is to use a participatory process to refine the ACE multifaceted implementation strategy. Strategy refinement through a participatory mapping approach will include development of (1) workflow processes to be used in ACE screening implementation and (2) the content of each implementation activity. The final product will be an implementation protocol to be used in the pilot trial that will include a logic model that delineates how implementation strategies are linked to implementation and effectiveness outcomes including specification of the mechanisms of action. The implementation phase will focus on testing the impact of the implementation strategy and ACE policy on primary and secondary outcomes using a SW-CRT design (aim 2).

**Fig. 2:**
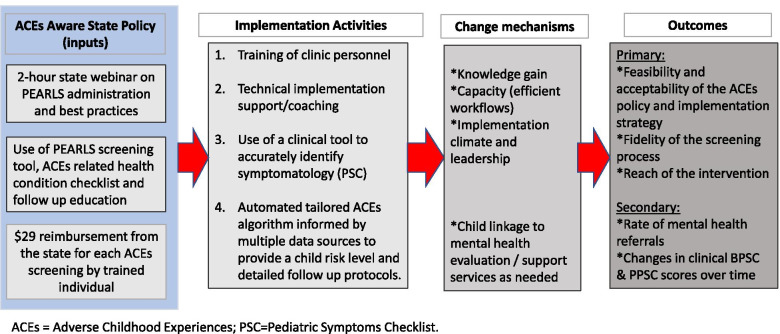
Overview of the ACE screening implementation study

Sustainment will focus on maintaining fidelity of policy implementation through the FQHC’s ongoing quality assurance efforts. In addition, this phase will focus on dissemination efforts to support scale-up across the FQHC system and additional FQHCs. Our partner is one of the largest FQHC systems in the country and with influence among peers and policy makers. Last, the study team will share study results with the California Office of the State Surgeon General for the ACEs Aware Initiative as a member of the review committee to promote the use of co-created and empirically tested strategies in the implementation of the ACEs Aware policy statewide. In sum, the resources provided by the ACEs Aware policy (inputs) of free online training, PEARLS tool, and screening reimbursement will be complemented by our proposed implementation activities designed to increase knowledge, capacity, and implementation climate and leadership. These conditions in turn will lead to achieving our proposed primary and secondary outcomes (Fig. 2).

## Study aims and approach

Aim 1: Use a participatory approach to refine a multifaceted implementation strategy to support the ACE screening policy in community-based clinics.

The core goals of the implementation strategy are to increase knowledge, ACE screening capacity, and efficient workflows at each clinic to address challenges faced by community-based settings in the implementation of a new intervention. Examples of potential challenges include limited opportunities for personnel training to gain intricate knowledge of ACEs, lack of ongoing intra-organizational support and buy-in during the implementation and sustainment phases, and inadequate use of technology to streamline workflows [31, 32].

The following four activities, informed by the mapping process, will address the core goals and used as the base during Implementation Mapping. While concrete strategy activities were identified a priori for grant proposal submission, these activities may be adapted and/or changed based on stakeholder engagement and other information obtained through the Implementation Mapping process (e.g., theory, empirical evidence, new data).

Activity 1: Short video-trainings for clinic personnel (care team staff and providers) on the administration of caregiver-reported screening tools through a trauma-informed care lens and implementation protocols. The trainings will complement the current state-sponsored 2-h on-line provider training and related trainings currently implemented at the FQHC.

Activity 2: Technical implementation support using a combined approach comprised of external academic consultants and internal FQHC personnel to increase its internal capacity. The FQHC champion (Director of Healthy Steps program), and others within the FQHC system, will receive training to support clinics as an implementation coach and provide hands-on support and monitoring to the clinical sites. Each clinic will receive intense implementation support from the FQHC implementation coach (about 1 h per week virtual or in person in the first 10-week trial crossover period) and less intense support in the succeeding 10-week periods (45–60 min every other week visit and phone consult). The fact that FQHC personnel will become trained implementation coaches will promote sustainment*.* In addition, the Trauma Informed Care (TIC) workgroup, comprised of FQHC leadership (i.e., clinical and quality departments), service providers, communications director, information technology (IT) coordinator, and academic collaborators, will support the implementation coach and provide ongoing buy-in within clinics. Research shows better results with a team implementation approach than with a single champion [38].

Activity 3: Use of a validated clinical screening tool—The Pediatric Symptoms Checklist is used in pediatric primary care settings to assess behavioral and social/emotional development [39, 40]. For this study, we will use the PSC tools that are tailored to children ages 0 to 5 years old. The PSC screening tool is needed, as the PEARLS only assesses ACE exposure and not mental health symptomatology, which is a common ACE sequalae [41].

Activity 4: Use of a technology-based tailored screening algorithm that will be pre-tested and refined before making it available to ACE screeners using computer tablets. The algorithm will be hosted in a HIPAA compliant REDCap (Research Electronic Data Capture) secure web application, hosted at the PI’s institution, to facilitate the screening process. For this pilot trial, the data will be collected in a separate system and transferred to the FQHC for documentation and reimbursement. As a next step after the completion of this pilot trial, the tested and refined ACE algorithm will be integrated into the FQHC’s EMR system.

### Use of a participatory approach to refine the ACE implementation strategy

We will use Implementation Mapping [42] a systematic and participatory process to refine strategies and increase the adoption, implementation, and sustainment of interventions in real-world settings [42], to develop and refine the implementation strategies described above and develop implementation protocols. A foundational principle of Implementation Mapping is community and stakeholder engagement. As such, the planning will take place with FQHC personnel, including members of the TIC workgroup (*n = 10*), research team (*n = 3*), and caregivers (*n = 8*). This methodology is expected to foster a co-created group process with shared responsibility for knowledge building and direction of the process [43, 44]. Earlier mapping steps may be re-visited to foster iterative feedback. This approach has been tested in health promotion programs [45] and to systematically “align interventions with consumer populations, regions, or contexts” [42]. We will convene three 90-min meetings with the TIC workgroup and a separate group with caregivers. Each workgroup session will be recorded and professionally transcribed. Each session transcript will inform the next mapping task.

Participants will provide their opinions (in English and Spanish) on how to improve the experience of caregivers and child patients when they visit the clinic and participate in ACE screenings; they also will offer suggestions on activities or strategies that can support clinic and provider efforts to implement ACE screenings. We will offer caregivers $50 compensation for their time at each mapping meeting they attend and the option to meet in the evening, weekends or other times convenient for caregivers. The expected outcome for this development phase is a co-created and refined protocol for an ACE implementation strategy and a refined ACE algorithm protocol.

### Steps to refine the ACE algorithm

**Fig. 3:**
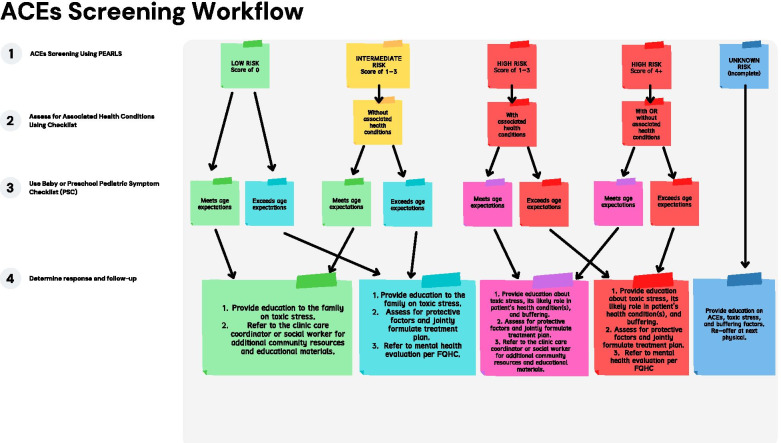
ACEs Aware 2019 pediatric ACE screening clinical workflow adapted algorithm for the study

Algorithms integrated into electronic systems support the successful translation of policy interventions into routine care, especially within fast-paced and under-resourced clinical settings [46]. As a first step, we will use computer tablets programmed with a secured web application to facilitate the screening process and pilot the screening algorithm. Figure 3 illustrates the state-sponsored ACE screening algorithm from July 2020. Blue circles and additional guidance have been added in the follow-up box for high risk level to refine the algorithm and prompt concrete action. A study database will include the PEARLS and the ACE-associated health conditions checklist provided by the state along with the study-added PSC measures. An algorithm will be programmed to score each measure and provide a category of child risk level (for potential trauma/toxic stress) that will be available to the physician and clinical staff. The child risk categories are: low risk if PEARLS score = 0 events endorsed and PSC scores = 0–2 (i.e., “appears to meet expectations”); intermediate risk if PEARLS score = 1–3 events endorsed, child does not have an ACE-associated physical condition and PSC scores = 0–2; and high risk if either PEARLS score = 1–3 events endorsed, child does not have an ACE-associated physical health conditions and PSC scores = 3+ (i.e., “further evaluation needed”) OR PEARLS score = 4+ events endorsed, child has an ACE-associated health condition and PSC scores = 3 +.

The ACE algorithm system will be tested prior to use in the pilot study to identify any problems and refine the system using two approaches. First, research team members and FQHC study co-leads/champions (i.e., data manager and Director of Health Steps programs) will try the system on their own and then convene to discuss glitches and provide suggestions for greater usability in clinics. Once approved by this group, one clinic will be selected (not a clinic for the trial study) to *beta* test the trial enrollment and data entry processes using the database and algorithm system. After the refinement and testing of the strategy activities during the 6-months developmental phase, we will finalize study materials and protocols. For aim 2, we will draw on what we learned from the developmental phase (aim 1) to inform a pilot study testing the feasibility, acceptability, fidelity and reach of the implementation strategy, and the impact of the ACE policy on child-level outcomes.

Aim 2: Pilot test the feasibility, acceptability, fidelity and reach of the implementation strategy, and the impact of the ACE policy on child patient-level outcomes.

## Methods

### Design

We will conduct a SW-CRT pragmatic trial without transition periods [36]. Recruitment of five clinics (clusters) serving 3000 children ages 0–5 years old will allow us to test the primary hypothesis of whether clinics employing a multifaceted implementation strategy will have higher fidelity to the ACE screening process and higher reach than other clinics. A secondary hypothesis will test whether the ACE policy will increase appropriate child-level mental health referrals and clinical symptom outcomes. The study will use mixed methods, with quantitative and qualitative sources of data to test the hypotheses, including child electronic health records, observations, interviews, and questionnaires with clinic personnel and caregivers. Following the SW-CRT design, each select clinic will receive the implementation strategy at different points in time and following a random order. Clinics will be selected to represent diverse geographic regions and counties, clinic size, pediatric patient volume, and patient-provider ratios.

**Fig. 4:**
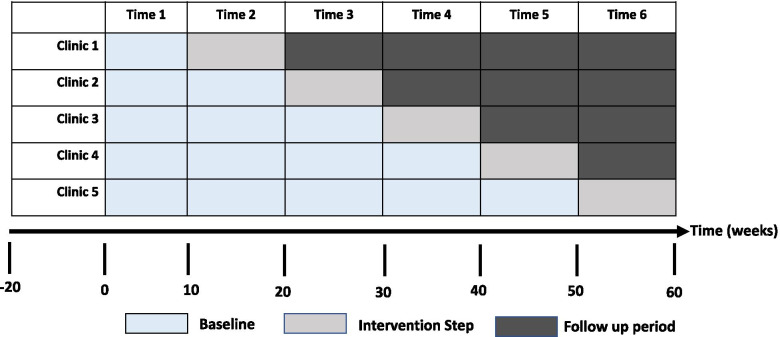
SW-CRT design and timing of data collection

The full pragmatic trial will last 15 months, conducted in six 10-week periods. Control status refers to clinics using standard care to identify children’s mental health needs through developmental screenings, discussions with providers during wellness visits, and/or following the ACE state protocol (i.e., PEARLS screenings, ACE health-related condition checklist, wellness exam and follow-up education). Intervention status refers to clinics implementing standard care in addition to the implementation strategy. During the baseline period (weeks 1–10), we will collect pre-implementation data for all five clinics on PEARLS screening rates, ACE-related health conditions checklist completion rates, mental health referral rates, and child socio-demographics. Figure 4 presents the SW-CRT design and timing of data collection.

This study design offers logistical and analytical advantages, including decreased burden for clinical sites, staggering of the implementation strategy with fewer resources needed such as the number of FQHC staff, steady allocation of resources and staff, and an opportunity to learn from each time period [35]. This approach will also provide a robust study of the ACEs Aware policy implementation, while accounting for the complex and dynamic nature of FQHC clinics and patient needs.

## Participants and procedures

### Participating clinics

The study statistician will select five clinics from a pool of 16 clinics providing family medicine, pediatric, or primary care for children ages 0 to 5 years old, located in Southern California and across the FQHC’s five regions to maximize variation in clinic characteristics (e.g., size, workforce) and patient characteristics, and external validity. If one of these clinics is unable to participate in the pilot at the particular time, an additional clinic will be selected from a pool of 8 additional eligible clinical sites. The regions span three large California counties and include urban, suburban, rural, and frontier communities. The clinics vary in size and resources and serve a diverse population. Random selection of the order of initiation of the intervention of clinical sites will avoid potential bias of selecting the most qualified/enthusiastic clinics to start the trial. The study statistician will generate the random allocation sequence and timing of crossover of clinics from control to intervention. The study PI and FQHC champions at the FQHC will invite the identified clinics via email to an informational video session to introduce the study and will email the clinic manager informational materials about the study. The FQHC study co-leads and the PI will introduce the study at a staff meeting in each clinic, including an endorsement from the TIC workgroup. Each selected clinic will sign an agreement to participate in the study before the trial starts.

### Caregiver/child

This study will gather information from caregivers (*n* = 900) on behalf of their child, following IRB approved informed consent and HIPAA releasing protocols, during the child’s wellness visit at the clinic. Caregiver eligibility is defined as the primary related adult, English and/or Spanish speaker, with permission to make decisions for the child and per clinic documentation. Consent for ACE screenings with caregivers will be part of their patient paperwork required as part of a wellness care visit at each clinic. Child data will be obtained from caregivers reporting on their child’s behalf (i.e., PEARLS and PSC). In addition, we will gather from each clinic in each 10-week period information on children’s socio-demographic characteristics, the number of ACE screenings completed, mental health referrals, and the number of eligible children using the FQHC’s EMR. The 0–5 year age group was selected because the FQHC is rolling out the ACE state policy in stages, with an initial focus on young children as a way of piloting the system and implementation efforts. A group of caregivers who participate in the ACE screenings (*n* = 25) will be contacted to participate in debriefing interviews to assess their satisfaction with the ACE screening experience for their child and solicit their suggestions for improvements. Given the current COVID-19 crisis, we will explore the potential impact of the pandemic as an adverse event for families. We will gather basic caregiver socio-demographics (e.g., sex, race/ ethnicity). Consented caregivers will receive $50 at the end of the interview as remuneration for their time and expertise.

### FQHC personnel

Clinic personnel who were involved in the implementation of the ACE screenings during the pilot trial (e.g., screeners, physicians, medical assistants, schedulers, clinic managers) will be invited to voluntarily participate in follow-up surveys (*n* = 20 individuals per clinic). FQHC co-leads will send a link to an online survey with a consent statement and option to accept or decline participation. Those who accept will complete surveys including basic demographic questions (e.g., age, gender, race and ethnicity, clinic role), questions related to implementation leadership and climate, and participants’ perceived support for ACE screenings in their clinic. Each participant will be given 30 days to complete their survey. Strategies used in previous research to increase response rates include email invitations distributed by the trained implementation coach, reminders during monthly meetings, including clear survey rationale, pre-notifications, and reminders [47]. Clinic personnel who participate in the implementation leadership and climate survey will be entered into $100 survey raffles as incentives. A smaller number of survey participants (*n* = 6 per clinic) will be randomly selected to participate in debriefing interviews. They will be asked their opinions on the feasibility of the ACE policy intervention, inner and outer context and bridging factor challenges, their satisfaction with the ACE implementation strategy, and suggestions for improvements. We also will ask questions about the financial aspects of the ACE policy implementation. They will receive $50 for their participation in the interviews.

### Description of the ACE screening intervention

The ACE screenings will be implemented at each clinic during the child’s health visit using the PEARLS survey, the PSC, and a checklist of ACE health-related conditions. Screenings procedures will be embedded into the clinics’ workflow: (1) FQHC study champion (i.e., data manager) identifies eligible children who are scheduled to attend the clinic on a given week; (2) the list of children and their chart number will be shared with the ACE screener 4–5 days prior to appointments; (3) screener will use the ACE computer tablet, programmed with the ACE algorithm, to consent the caregiver and to enter data; (4) the system will provide each risk category (low/intermediate/high) and suggested follow-up action based on that category; and (5) the physician uses the data to discuss with the family and inform clinical decision-making and follow-up action.

### Measures and trial outcomes

#### PEARLS

This is a caregiver-reported tool (17-items) designed to identify exposure to childhood adversity and stress factors that may lead to toxic stress and negative health outcomes. The number of endorsed events are summed up (0–4+) to identify risk level for toxic stress and potential symptomatology. The PEARLS is available in English and Spanish. It will be collected at baseline and at each time period. The tool has high face validity with validation phase comprised of cognitive interviews and item wording refinement with parents/caregivers, physicians, and clinic staff [6, 48].

#### ACE-related health conditions checklist

This state-sponsored list includes 25 ACE-associated health conditions [18]. Physical conditions include asthma, allergies, and unexplained somatic symptoms such as headaches. Mental health related conditions include aggression, depression, ADHD, and anxiety. This information will be obtained from the FQHC’s EMR system. Data will be collected at baseline and at each time period.

#### Pediatric Symptoms Checklist (PSC)

This is a caregiver-reported tool measuring children’s psychosocial functioning, which assesses externalizing, internalizing, attention problems, and parenting challenges [40]. Parents select responses as “not at all” = 0, “somewhat” = 1, “and very much” = 2. A score of 9+ indicates child is “at risk and needs further evaluation.” Measure is taken at each time period of the pilot trial. See trial outcomes in Table 1.

**Table 1:** ACE screening trial outcomes

Outcome	Measurement	Data source	Frequency^**a**^
*Clinic level outcomes (primary)*			
**Reach of ACE screenings**	Proportion of eligible children participating in ACE screenings. We expect between 80 and 92% of eligible children will be screened; based on pediatric screening studies in primary care [49, 50]	FQHC EMR system	Week 10 of each time period
**Mental health referral rates**	Number of mental health referrals (behavioral analysis, behavioral health, care coordinator, care management, child development center or social work) divided by the total # of eligible children. For children deemed at high risk for toxic stress and/or mental health needs. Expect 11.4% increased referral rate based on a similar study [29] and using current referral rate of 3.8% to inform this threshold and per FQHC i2itracks report	FQHC EMR system	Week 10 of each time period
*Clinic level outcomes (secondary)*			
**Implementation feasibility** [6]	Self-reported 4-item survey evaluating feasibility of implementation efforts. 4-pt Likert scale; average score of 4+ shows ACE policy and implementation strategy perceived as feasible. Good internal consistency (*α*=0.89). Test-retest reliability *r* = 0.88	FQHC personnel	Week 10 of the intervention time period
**Implementation acceptability** [6]	Self-reported 4-item survey evaluating acceptability of ACE policy and implementation efforts. 4-pt Likert scale; average score of 4+ shows acceptability. Good internal consistency (*α* = 0.83). Test-retest reliability *r* = 0.83		Week 10 of the intervention time period
**Fidelity**	Checklist assessing adherence to ACE screening protocols and competence of performance. Deviations/concerns will be documented and immediately reported back to clinic personnel. We expect at least 67% fidelity (number of endorsed deviations/all items in the checklist) based on a previous study [49]. Adaptations and emerging challenges will be documented and reported to the research team. Observation checklists/audits are effective strategies to improve fidelity of performance [51]	Implementation coach	Weeks 5 and 10 of each time period
*Child/parent level outcomes*			
**Changes in PSC scores**	Mean score differences from eligible screened children who were deemed at high or at intermediate risk.	Randomly selected group of caregivers	10 weeks after child’s ACE screening
*Mediators*			
**Implementation leadership** [48]	12-item survey comprised of four subscales measuring proactive leadership, knowledgeable leadership, supportive leadership, and perseverant leadership. Strong reliability for the total scale (*α* = 0.98). An average score of 4+ will be used as threshold; 5-point Likert scale (not at all-very great extent). Subscale score is based on the mean score for the items; total score is the mean of the subscale scores [48]	Clinic personnel	Week 7—intervention period—and week 9—every other time period
**Implementation climate** [52]	6-item survey measuring the strategic climate for the implementation of interventions. Items are rated on a 5-item Likert scale (completely disagree-completely agree)		Week 7—intervention period—and week 9—every other time period
*Other measures*			
**Child socio-demographic characteristics**	Variables include sex, self-identified race and ethnicity, age, language of preference for health care receipt, born in the USA. Note: EMR system does not report data on caregivers of child patients	EMR system	Week 10 of each time period

^a^Within a 10-week time period. The SW-RCT is comprised of six 10-week time periods: baseline, intervention, and four follow-up periods, depending on clinic schedule

All data will be shared between the PI and partner FQHC through password-protected de-identified files that will be saved on a secured network at the PI’s institution to minimize risks.

### Sample size and power calculations

The clinical trial was powered using the outcome measure of ACE screening “reach” using the approach as described in Hussey and Hughes [49]. Power calculations were estimated for this pilot trial that is aiming to assess the feasibility and acceptability of the ACE implementation strategy and establish an effect size for the achieved ACE screening rate (trial reach) to be used for planning a larger definitive implementation and effectiveness trial. Selected clinics will offer sufficient flow of wellness child visits per month and number of service providers to reach the estimated sample size. The analysis assumes that the 5 clinics will implement the intervention with 6 steps (time periods lasting 10 weeks each) and the same minimum number of children per clinic will be seen during each step. The coefficient of variation for the generalized linear mixed model (GLMM) to be used in the analysis is assumed to be 0.5. Using summary data provided by our health care partner, an estimated 59 child health visits will occur at each clinic per 10-week time period. We used this sample size of 59 per clinic per trial step (*n* = 1770 total well-child visits), as well as a more conservative sample size of 30 per clinic per step (*n* = 900 total well-child visits), to estimate the minimum detectable differences in ACE screening rates at a power of 80% and testing at a 2-sided alpha = 0.05.

The minimum detectable differences (comparing ACE screening implementation intervention to control periods) was calculated based on a range of completed ACE screenings in the control periods. As indicated in Table 2, the projected sample size of 900 total well-child visits is sufficient to detect rate differences of 2–9.1% depending on the screening rate in the control periods. A more conservative sample size of 30 per clinic per step will still allow detection of rate differences of at least 2.7–13%.

**Table 2:** Minimum detectable differences in ACE screening rates in intervention vs control periods, 80% power

Sample size per clinic per time period	ACE screening rates in control time periods				
	1%	5%	10%	15%	20%
59 (total = 1170)	2%	4.9%	6.8%	8.2%	9.1%
30 (total = 900)	2.7%	6.6%	9.5%	11.5%	13%

### Analysis plan and reporting guidelines

All analyses will control for child socio-demographic characteristics (i.e., race, ethnicity, age, gender). Trial reporting follows the CONSORT 2010 cluster randomized trials guidelines, with extension to stepped-wedge cluster randomized trials [51], the SPIRIT guidelines, and the Standards for Reporting Implementation Studies (StaRI) guidelines.

### Analysis of quantitative data

Descriptive statistics will use median (IQR) for continuous and frequency (percent) for categorical variables. Numbers of eligible children will be summarized by intervention versus control periods and average numbers of children per cluster/clinic will be summarized by period. We will examine the effect of the ACE screenings and implementation strategy on the intervention’s reach by using a generalized linear mixed model (GLMM) approach [49] to model clustering of participants within clinics. We will use an intent-to-treat approach to estimate the magnitude of treatment effect for the primary outcome reach (ACE screening rate). Analysis of stepped-wedge trial data must also consider possible confounding due to correlation of the treatment intervention with calendar time (as more clinics change to the intervention period over calendar time). The mixed effects models will therefore include a random effect for cluster (clinic) and fixed effects for intervention (0 for control and 1 for implementation periods) and time period (to control for calendar time). For the outcome of reach (whether screening occurred), the model linear predictor is linked to the event of a child’s screening (binary dependent variable) through a logit link function. This logistic GLMM will test for differences in screening rates (reach) in intervention compared to control periods; the intervention effect (i.e., difference in screening rates) with 95% confidence interval will be reported. Model-based estimates of probability of screening (i.e., screening rate/reach) will be reported in intervention and control periods, with 95% confidence intervals. The intra-cluster (within clinic) correlations as well as the time effect (main effect of time period) will be reported and used for future trial planning.

In a subgroup analysis, we will also test for a differential effect on outcomes by child socio-demographic characteristics (e.g., gender) by adding an intervention-gender product term. We will report the magnitude of missing data and compare demographic, clinical characteristics, and outcomes between observations with versus without missing data. In sensitivity analyses, we will perform multiple imputations using fully conditional specification to obtain 10 complete datasets; summary estimates of intervention effects with 95% confidence limits over the 10 datasets will incorporate within- and between-dataset sampling variability [49, 50]. The secondary outcome of mental health referral rates will be analyzed using the same approach.

Clinic outcome variables of implementation feasibility, acceptability, and fidelity will be analyzed using descriptive statistics. The internal consistency of all variable scales will be calculated using Cronbach’s alphas.

Child-level outcome of changes in PSC scores will be analyzed using GLMMs, with each child contributing pre- and post-PSC scores and specifying a Gaussian distribution with an identity link function. An additional random intercept at the child level (nested within clinic) will be included in the GLMM. An indicator variable for assessment time (0 for pre-, 1 for post-test) and an interaction of intervention by assessment time will test whether the mean PSC change differs in intervention vs control periods.

### Analysis of qualitative data

All interviews will be audio-taped and professionally transcribed. Data analysis will include deductive coding guided by results of the Implementation Mapping process and EPIS principles. Transcripts will be coded to identify common themes, capture analytical categories from the emerging themes, and depict associations between categories. Development of codes will begin with, but not be limited to, the EPIS framework dimensions and constructs. We will also use Implementation Mapping principles to identify codes for implementation tasks (performance objectives), factors influencing implementation (determinants), potential methods, and practical applications of those methods (feedback on implementation strategies used). Interviews will be transcribed using pseudonyms to increase confidentiality. Two trained coders (postdoc and graduate student) will analyze data. Inter-rater reliability (IRR) will be calculated as the proportion of agreed codes over the total number of codes in the document [52]. We will calculate overall IRR (between coders) using Kappa statistics. Rigor strategies will include documenting coding decisions made by the research team, and tracking thematic development [53]. A detailed codebook will be developed based on the EPIS framework. The quantitative information from survey data (e.g., feasibility, acceptability) will be aligned with the emerging themes from the qualitative survey data with the use of matrices tables.

### Additional analyses of process measures

An exploratory approach we might pursue to better understand the role of the ACE policy intervention on the measured outcomes is whether this association is mediated through a clinical context (inner context) that is supportive of innovation (i.e., implementation leadership and climate). We will use structural equation modeling with bootstrapping to detect a signal on the mediational effects of implementation leadership and climate on the feasibility, acceptability, fidelity, and reach of the ACE implementation strategy. We hypothesize that meeting implementation outcomes are a function of (a) the implementation leadership characterized by proactive action, knowledge, support, and perseverance [54] and (b) overall climate for implementation related to perceived work settings promoting needed skills, incentives and reduced challenges to influence innovation use [55].

## Discussion

This project capitalizes on a rare opportunity to pilot test an implementation strategy to evaluate the impact of a state-wide policy intended to improve child behavioral health in FQHC settings. This research is significant for its potential to investigate a critical care gap and examine whether a tailored implementation strategy of an ACE screening policy increases mental health referrals and improves care for children exposed to adverse events and deemed at high risk for toxic stress and clinical symptomatology. As such, the testing of strategies that can successfully embed ACE screenings into primary care is urgently needed.

The overall goal of this study is to bring together researchers, practitioners, and patients to co-create an implementation strategy and examine its impacts on ACE screening fidelity and reach, rates of child mental health referrals, and clinical scores. In addition, the study will assess feasibility and acceptability of the implementation strategy and mechanisms including implementation leadership and climate.

Upon completion of this project, we expect to have tested the feasibility of the ACE intervention and implementation strategies within one of the largest FQHC system in the USA [56]. This study will use participatory methodology and engage diverse stakeholders that include FQHC management, IT, EMR, behavioral health, quality, service providers, research department, and caregivers as well as a diverse team of researchers. Our goal is to promote a co-created process for the development of implementation protocols that includes input from all stakeholders through a meaningful participatory process.

We seek to increase the capacity of the FQHCs’ inner context to successfully implement healthcare policies benefitting their pediatric population. Knowledge from this study can ultimately contribute to a meaningful public health impact of the ACE policy in California and nationwide. This is a relevant area of research given that systematic approaches testing the effectiveness of implementation strategies designed to support pediatric screenings in primary care are yet to be developed. Last, this study can strengthen implementation science given the lack of studies testing strategies during a policy’s implementation process [57].

## Data Availability

The complete protocol can be accessed by contacting the PI and corresponding author.

## Abbreviations

ACEs: Adverse childhood experiences
FQHCs: Federally Qualified Health Centers
PEARLS: Pediatric ACEs and Related Life-events Screener
HIPAA: Health Insurance Portability and Accountability Act
PSC: Pediatric Symptoms Checklist
PI: Principal investigator in this study
AAP: American Academy of Pediatrics
TIC: Trauma Informed Care
Medicaid: Type of managed healthcare plan in the State of California, USA, for people with low or no income
EMR: Electronic medical records
